# Supplementary material for: Demonstrating the Applicability of Smartwatches in PM2.5 Health Impact Assessment
Source: Sensors (Basel). 2021 Jul 4;21(13):4585. doi: 10.3390/s21134585 (PMC8271904; doi:10.3390/s21134585)
Supplement: Supplementary file 1 [file sensors-21-04585-s001.zip › sensors-1242417-supplementary.pdf]

sensors

Article

Demonstrating the Applicability of Smartwatches in $PM_{2.5}$ Health Impact Assessment

Ming-Chien Mark Tsou [1], Shih-Chun Candice Lung [1,2,3,*] and Chih-Hui Cheng [1]

[1] Research Center for Environmental Changes, Academia Sinica, Taipei 115, Taiwan; marktsou09@gate.sinica.edu.tw (M.-C.M.T.); chihhui104@gate.sinica.edu.tw (C.-H.C.)
[2] Department of Atmospheric Sciences, National Taiwan University, Taipei 106, Taiwan
[3] Institute of Environmental and Occupational Health Sciences, National Taiwan University, Taipei 100, Taiwan
* Correspondence: sclung@rcec.sinica.edu.tw; Tel.:+886-2-27875908; Fax: 886-2-27833584

Supplementary Figure Legend

Supplementary Figure S1. The activity intensity measurements obtained from AS-LUNG-P during sleeping periods. The dash line indicates 1100 mG.

Supplementary Figure S2. The correction equation for converting the activity intensity measurements obtained from AS-LUNG-P to RootiRx-comparable measurements, after excluding data below 1100 mG.

Citation: Tsou, M.-C.M.; Lung, S.-C.C.; Cheng, C.-H. Demonstrating the Applicability of Smartwatches in $PM_{2.5}$ Health Impact Assessment. *Sensors* **2021**, *21*, 4585. https://doi.org/10.3390/s21134585

Academic Editor(s):

Received: 15 May 2021
Accepted: 30 June 2021
Published: 4 July 2021

Publisher's Note: MDPI stays neutral with regard to jurisdictional claims in published maps and institutional affiliations.

Copyright: © 2021 by the authors. Licensee MDPI, Basel, Switzerland. This article is an open access article distributed under the terms and conditions of the Creative Commons Attribution (CC BY) license (http://creativecommons.org/licenses/by/4.0/).

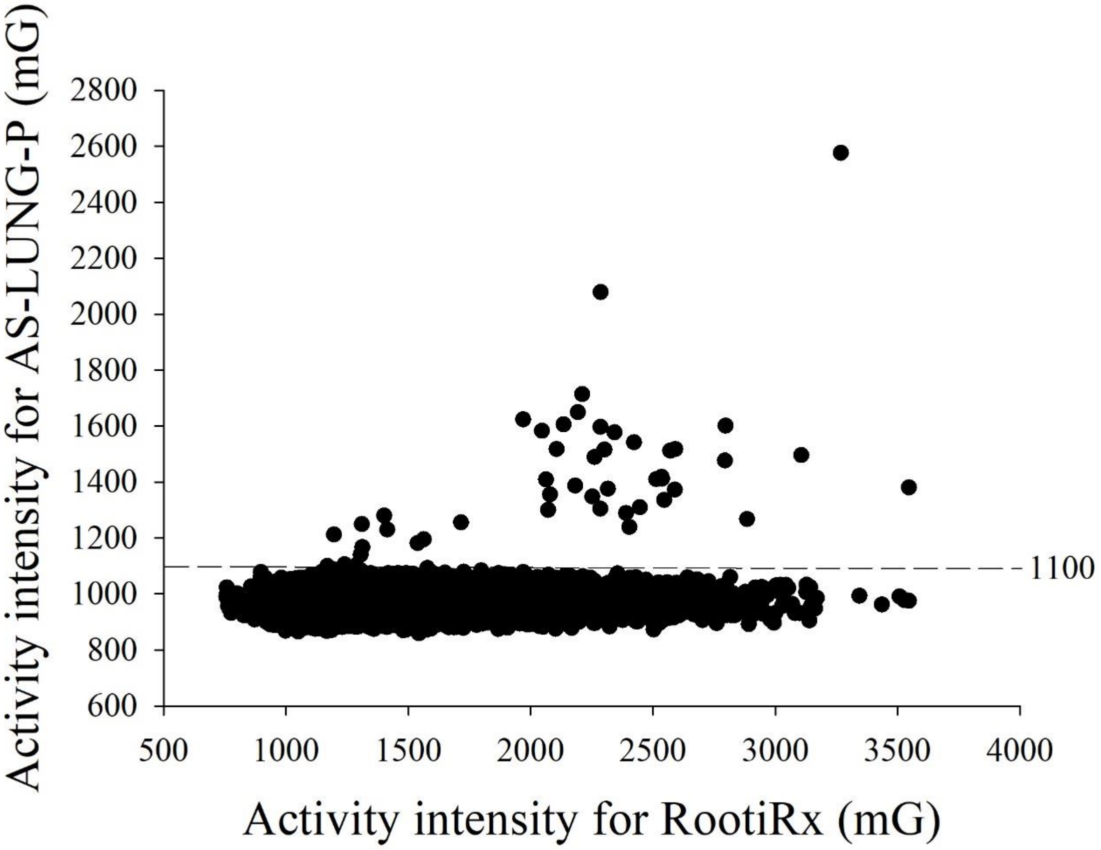

Figure S1. The activity intensity measurements obtained from AS-LUNG-P during sleeping periods. The dash line indicates 1100 mG.

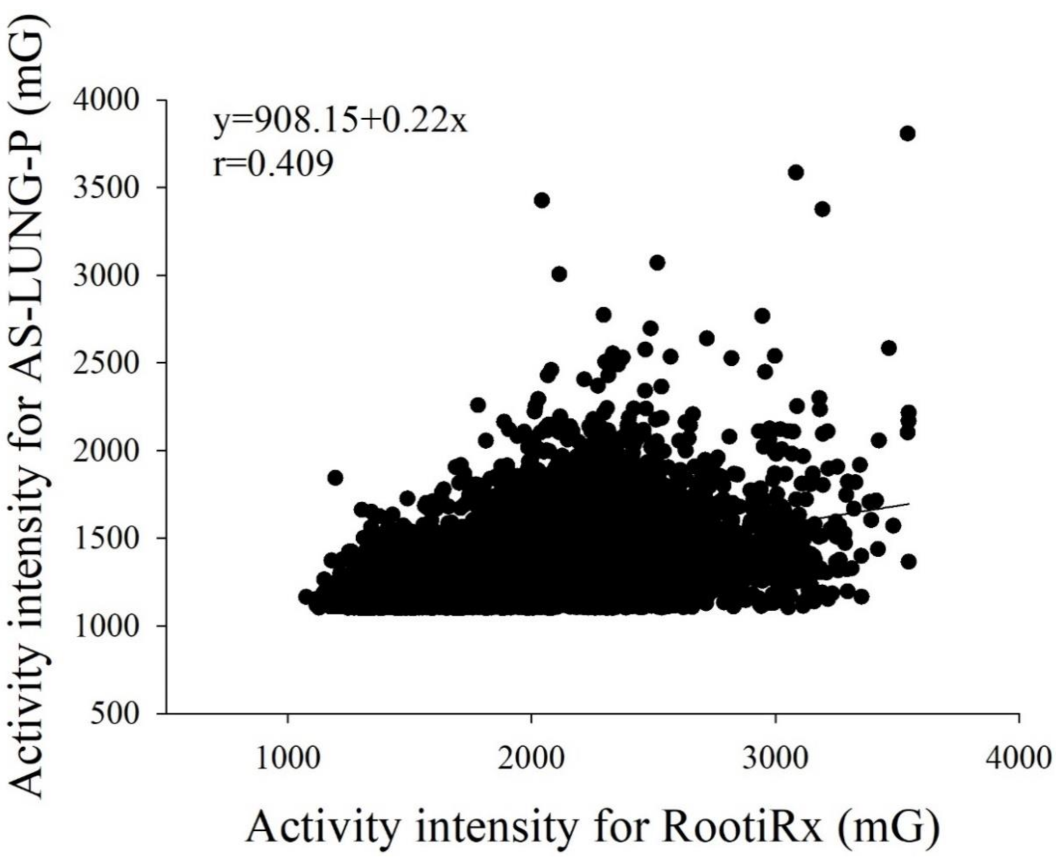

Figure S2. The correction equation for converting the activity intensity measurements obtained from AS-LUNG-P to RootiRx-comparable measurements, after excluding data below 1100 mG.
